# Supplementary material for: Consumers’ psychological constructs regarding hybrid meat products: A scoping review protocol
Source: PLoS One. 2026 Feb 18;21(2):e0343059. doi: 10.1371/journal.pone.0343059 (PMC12915932; doi:10.1371/journal.pone.0343059)
Supplement: S2 Appendix — (DOCX) [file pone.0343059.s002.docx]

# **S2 - Appendix 2 - Search Strategy**

| **Database** | **Key words and query** | **Preliminary search results** |
| --- | --- | --- |
| PubMed | ((meat substitutes[MeSH Terms]) OR ''meat substitute* OR "hybrid burger*" OR "meat analog*" OR "hybrid meat" OR "hybrid protein*" OR "hybrid patty" OR "hybrid meat product*" OR "hybrid sausage*" OR "alternative meat" OR "blended meat" OR "meat hybrid*" OR "restructured meat" OR "partial meat replacement*" OR "plant-meat blend*") | 4,607 |
| EBSCOhost (APA PsycInfo, APA PsycArticles, Psychology and Behavioral Sciences Collection, CINAHL, Global Health, GreenFILE and MEDLINE) | (''meat substitute* OR "hybrid burger*" OR "meat analog*" OR "hybrid meat" OR "hybrid protein*" OR "hybrid patty" OR "hybrid meat product*" OR "hybrid sausage*" OR "alternative meat" OR "blended meat" OR "meat hybrid*" OR "restructured meat" OR "partial meat replacement*" OR "plant-meat blend*") | 5,728 |
