# Supplementary material for: Consumers’ psychological constructs regarding hybrid meat products: A scoping review protocol
Source: PLoS One. 2026 Feb 18;21(2):e0343059. doi: 10.1371/journal.pone.0343059 (PMC12915932; doi:10.1371/journal.pone.0343059)
Supplement: S3 Appendix — (DOCX) [file pone.0343059.s003.docx]

# **S3 - Appendix 3 - Data Extraction Form**

| **Item** | **Description** |
| --- | --- |
| Study ID | Authors and publication year |
| Country | Country or countries in which the study is conducted |
| Study type | Type of study design (e.g., qualitative, quantitative, experimental, observational) |
| Aim | Purpose or research question(s) of the study |
| Participants | Description of the study population, including key demographic characteristics (e.g., age range, gender distribution, sample size), relevant inclusion/exclusion criteria, and any specific subgroups targeted (e.g., consumers, patients, students). |
| Product type | Specific type of hybrid meat or related product studied |
| Psychological construct | Psychological factors or constructs investigated (e.g., attitudes, perceptions, intentions) |
| Findings | Key results and conclusions relevant to the review topic |
| Indicated research gaps | Gaps or limitations identified by the study for future research |
