# Supplementary material for: Consumers’ psychological constructs regarding hybrid meat products: A scoping review protocol
Source: PLoS One. 2026 Feb 18;21(2):e0343059. doi: 10.1371/journal.pone.0343059 (PMC12915932; doi:10.1371/journal.pone.0343059)
Supplement: Attachment — Submitted filename: Response to Reviewers.docx [file pone.0343059.s004.docx]

Response to Reviewers

Dear Reviewers,

Thank you very much for your feedback. We hereby respond to the points raised:

1. **Please ensure that your manuscript meets PLOS ONE's style requirements, including those for file naming. The PLOS ONE style templates can be found at** <https://journals.plos.org/plosone/s/file?id=wjVg/PLOSOne_formatting_sample_main_body.pdf> and <https://journals.plos.org/plosone/s/file?id=ba62/PLOSOne_formatting_sample_title_authors_affiliations.pdf>
   We have rechecked the PLOS ONE style requirements and confirm that we fully meet the PLOS ONE’s style requirements now.
2. **When completing the data availability statement of the submission form, you indicated that you will make your data available on acceptance. We strongly recommend all authors decide on a data sharing plan before acceptance, as the process can be lengthy and hold up publication timelines. Please note that, though access restrictions are acceptable now, your entire data will need to be made freely accessible if your manuscript is accepted for publication. This policy applies to all data except where public deposition would breach compliance with the protocol approved by your research ethics board. If you are unable to adhere to our open data policy, please kindly revise your statement to explain your reasoning and we will seek the editor's input on an exemption. Please be assured that, once you have provided your new statement, the assessment of your exemption will not hold up the peer review process.**
   All available data will be made publicly available on the Open Science Framework. We have received a confirmation on January 26, 2026 from Plos ONE that the information was updated on our behalf. We have received the instruction to not include it in the Manuscript itself as the data availability statement will be published alongside our manuscript should it be accepted for publication.
3. **Please include captions for your Supporting Information files at the end of your manuscript, and update any in-text citations to match accordingly. Please see our Supporting Information guidelines for more information:** [**http://journals.plos.org/plosone/s/supporting-information**](http://journals.plos.org/plosone/s/supporting-information)**.**

We have added the heading *“Supporting Information”* to the manuscript. Furthermore, we have updated the captions of the supporting materials to S1 Appendix: PRISMA-P 2015 Checklist, S2 Appendix: Search Strategy, and S3 Appendix: Data Extraction Form. We also updated the in-text citations accordingly to refer to S1 Appendix, S2 Appendix, and S3 Appendix.

4. **We are unable to open your Supporting Information file “PRISMA-P checklist - seperate file.docx”. Please kindly revise as necessary and re-upload.**
   The updated PRISMA P-checklist is now attached in PDF format in the appendix.
5. **If the reviewer comments include a recommendation to cite specific previously published works, please review and evaluate these publications to determine whether they are relevant and should be cited. There is no requirement to cite these works unless the editor has indicated otherwise.**
   Not applicable.
6. **Please review your reference list to ensure that it is complete and correct. If you have cited papers that have been retracted, please include the rationale for doing so in the manuscript text, or remove these references and replace them with relevant current references. Any changes to the reference list should be mentioned in the rebuttal letter that accompanies your revised manuscript. If you need to cite a retracted article, indicate the article’s retracted status in the References list and also include a citation and full reference for the retraction notice.**
   The reference list has been reviewed and no additional changes were made.

**Reviewer #1:**

Dear Authors,

- **The manuscript presents a well-defined scoping review protocol, addressing a current and relevant topic in light of the environmental and health challenges associated with meat consumption. The introduction is clear, well-structured, and contextualizes hybrid meat products as a promising strategy for transitioning to more sustainable dietary patterns, demonstrating theoretical consistency and good articulation with existing literature.**
  Thank you very much.
- **As suggestions for methodological improvement, in the methods section, note that the manuscript presents separate subtopics, such as research question, inclusion and exclusion criteria, and types of sources. We suggest that these contents be kept integrated into the larger topic "Methods," in accordance with the recommendations of the Joanna Briggs Institute.**
  Thank you very much for your suggestions. We changed this as suggested.
- **Furthermore, the content currently presented in the subtopic "Types of sources" could be directly incorporated into the inclusion criteria, contributing to greater clarity and organization of the text.**
  We have deleted the sub-heading ‘types of sources’, so that this section is directly incorporated into the inclusion criteria.
- **It is also recommended that the manuscript include at least the main descriptors used in a standardized way in the search strategies, even if the complete strategies are available in supplementary material, in order to strengthen the transparency and reproducibility of the protocol.**

In order to strengthen the transparency and reproducibility of the protocol the following sentences have been added to the text:

*‘’The Boolean operator OR was used to combine synonymous terms and closely related concepts within the search strategy.’’

‘’MeSH terms were used where available to improve search precision.’’

‘’A cross-database adoption strategy will be used to ensure a comprehensive and systematic identification of relevant literature.’’*

- **Finally, it is suggested to include page numbering corresponding to the items of the PRISMA-P checklist, facilitating the monitoring of compliance with reporting guidelines.**
  This is done in the updated PRISMA P-checklist attached in the appendix. See also our response to the editor.
- **Overall, this is a methodologically consistent protocol, well aligned with international guidelines for scoping reviews and with the potential to offer a relevant contribution to the field. With minor adjustments to the organization and presentation of the method, the manuscript is likely to be further strengthened.**
  Thank you very much.

**Reviewer #2:**

- **The use of AI is explicitly stated, which is positive in terms of transparency. However, the description is insufficiently detailed to guarantee reproducibility: It does not explain how ChatGPT was used (general prompt, iterative refinement, validation by experts). Furthermore, it is unclear whether the terms suggested by the AI were** **checked against controlled vocabularies (MeSH, APA Thesaurus, CINAHL Headings).**
  Thank you very much for your feedback. We have detailed the description of using ChatGPT by including the following text:

*‘’To supplement this process, ChatGPT was consulted to identify additional terminology and variations associated with hybrid meat products, using the prompt ‘Generate database-appropriate synonyms and related terms for “hybrid meat” that can be used in a search string for both PubMed and EBSCOhost.’. The proposed synonyms were reviewed by the research team and incorporated into the search strategy where appropriate. New terms were cross-checked against MeSH.’’*

- **There is a potential incongruity: a preliminary search in June 2025 and a final search scheduled for January 2026. It would be important to clarify whether the strategy will be updated or revalidated before the final search, especially considering the use of emerging terms in an innovative field such as hybrid foods.**
  Thank you very much for bringing this point to our attention. The strategy remained unchanged for the final search, as we believe it remains current and appropriate for the field. For clarity, we have added the following sentence to the manuscript: ‘’*The preliminary search strategy will be applied without modification during the final search as it remained appropriate.’’*
- **Although it is mentioned that the complete strategy is in Appendix 2, the main text could indicate: the use of Boolean operators; the searched fields (ti, ab, kw); and the cross-database adaptation strategy.**

The following sentences have been added to the manuscript for further clarification:

*‘’The Boolean operator OR was used to combine synonymous terms and closely related concepts within the search strategy.’’

‘’MeSH terms were used where available to improve search precision.’’

‘’A cross-database adoption strategy will be used to ensure a comprehensive and systematic identification of relevant literature.’’*
